# Single-Crystal-to-Single-Crystal Anion Exchange in a Gadolinium MOF: Incorporation of POMs and [AuCl_4_]^−^

**DOI:** 10.3390/polym8050171

**Published:** 2016-04-26

**Authors:** Javier López-Cabrelles, Guillermo Mínguez Espallargas, Eugenio Coronado

**Affiliations:** Instituto de Ciencia Molecular (ICMol), Universidad de Valencia, c/Catedrático José Beltrán, 2, Paterna 46980, Spain; Javier.Lopez-Cabrelles@uv.es (J.L.-C.); eugenio.coronado@uv.es (E.C.)

**Keywords:** MOFs, anion exchange, polyoxometalates

## Abstract

The encapsulation of functional molecules inside porous coordination polymers (also known as metal-organic frameworks, MOFs) has become of great interest in recent years at the field of multifunctional materials. In this article, we present a study of the effects of size and charge in the anion exchange process of a Gd based MOF, involving molecular species like polyoxometalates (POMs), and [AuCl_4_]^−^. This post-synthetic modification has been characterized by IR, EDAX, and single crystal diffraction, which have provided unequivocal evidence of the location of the anion molecules in the framework.

## 1. Introduction

Coordination polymers, composed of metal ions or clusters connected by organic ligands into crystalline networks [1,2] are ideal systems for the design of functional materials. The last 30 years have witnessed an expansion in the study of coordination polymers since the pioneering work by Robson [3,4] Metal-organic frameworks (MOFs) [4,5,6,7,8,9,10,11,12,13,14,15,16,17,18,19,20,21] are an important subset of coordination polymers with enormous internal surface areas due to their porous structures, and have shown great versatility in the design of crystalline materials with a wide range of properties, including gas storage [22,23,24,25,26], chemical separation [27,28,29], catalysis [30,31,32,33,34], bio-applications [35], or magnetism [36], among others.

A current strategy to further incorporate additional functionalities on MOFs involves their post-synthetic modification [37,38,39,40,41,42]. This can be achieved either in a covalent manner, *i.e.*, through post-modification of organic ligands via chemical reactions with minor changes in the connectivity of the metal centers, for which examples are scarce [43], or through encapsulation of guest molecules into the empty space of MOFs [44]. An extensively studied non-covalent post-synthetic modification of coordination polymers consists of ion exchange [45,46,47,48,49,50,51,52,53,54,55,56,57], which has shown to be an effective way to tune gas sorption [58,59] or to serve as anion recognition in more complex smart-materials [60]. Very recently, this approach has also been used in order to immobilize cationic catalysts [61,62,63]. Most commonly, the ions are trapped in the pores and are weakly bonded to the (ionic) framework, thus facilitating the exchange process, although metal-bound anions have also been proved to undergo this kind of process [64]. These ionic MOFs are attracting significant attention because of the possible improvement of host–guest interaction, as the presence of ions inside the channels of the framework can be utilized for specific interactions with various incoming guest molecules [65].

Polyoxometalates (POMs) are bulky anions with electronic versatility that have a wide range of physical and chemical properties, which make them suitable for multiple applications in the fields of chemistry, materials, molecular nanoscience, or biology, among others [66,67]. Their incorporation into the channels of MOFs is a promising approach to avoid agglomeration of these active species causing thus an enhancement in surface area and accessibility to active sites [68]. Furthermore, this approach can lead to the combination of organic and inorganic catalytic components synergistically [69]. The incorporation of POMs has been typically achieved through the use of POMs as templates for the MOF formation [68,69,70,71,72,73], although post-synthetic incorporation through impregnation [74,75,76,77] or via anion exchange [78] has been also shown successful.

Here we present the preparation of a Gd-based MOF of formula [Gd(bipyNO)_4_]_3_(TfO)_9_·Χ solvent (bipyNO = 4,4′-bipyridyl-*N,N′*-dioxide; TfO = triflate), and its anion-exchange capability with POMs and AuCl_4_^−^, in order to analyze the effects of charge and size in the successful incorporation in the internal cavities. In these cases, the anion exchange process occurs in a single-crystal-to-single-crystal manner thus providing structural evidence of the location of the encapsulated species.

## 2. Materials and Methods

### 2.1. Synthesis of **1**

Gadolinium triflate hydrate (0.05 mmol) was carefully covered with dichloromethane (CH_2_Cl_2_, 8 mL). Then, a layer of pure MeOH (8 mL) was stacked slowly, and on top of this a MeOH solution (17 mL) containing 4,4′-bipyridine-*N*,*N*′-dioxide (bipyNO, 0.18 mmol; 37.1 mg) previously dissolved. After three days crystals suitable for single-crystal X-ray diffraction appeared, [Gd(bipyNO)_4_]_3_(TfO)_9_. The crystalline material was not stable in air as they were quickly desolvated and collapsed. Thus, they were stored in MeOH solution, and thermogravimetric analyses could not be performed.

### 2.2. Anion Exchange

Crystals of the different polyoxometalates, (TBA)_2_[Mo_6_O_19_], (TBA)_2_[W_6_O_19_], (TBA)_3_[W_5_VO_19_], (NHex_4_)_2_[SMo_12_O_40_] and the discrete metal-organic specie, NaAuCl_4_, were synthesized as previously reported [79,80,81]. The anion exchange processes were carried out by dissolving *ca.* 50 mg of the corresponding salt in acetonitrile (5 mL) yielding in all cases a clear solution. Then, single crystals of compound **1** (1 mg) were added to each solution, and the color of the crystals began to change immediately to yellow (**1–Mo_6_O_19_** and **1–AuCl_4_**). The mixed compound **1–W_6_O_19_–AuCl_4_** was obtained in a two step process: first, single crystals of compound **1** (1 mg) were added to a solution containing [AuCl_4_]^−^, resulting in **1–AuCl_4_**; secondly, this solid, **1–AuCl_4_**, was immersed in a solution containing [W_6_O_19_]^2−^ anions.

Single crystals of [Gd(bipyNO)_4_]_3_(Mo_6_O_19_)_3_(TfO)_3_·solvent (**1–Mo_6_O_19_**), [Gd(bipyNO)_4_]_3_(W_6_O_19_)_3_(TfO)_3_·solvent (**1–W_6_O_19_**), [Gd(bipyNO)_4_]_3_(AuCl_4_)_7.5_(Cl)_1.5_·solvent (**1–AuCl_4_**), [Gd(bipyNO)_4_]_3_(W_6_O_19_)_2.25_(AuCl_4_)_1.5_(Cl)_3_·solvent (**1–W_6_O_19_–AuCl_4_**), were analyzed by single-crystal diffraction in addition to IR and EDAX (solvent = mixture of MeOH and CH_2_Cl_2_).

#### Single crystal diffraction

Single crystals of all compounds were mounted on glass fibers using a viscous hydrocarbon oil to coat the crystals. X-ray data were collected at 120 K on a Supernova diffractometer equipped with a graphite-monochromated Enhance (Mo) X-ray Source (λ = 0.71073 Å). The program CrysAlisPro, Oxford Diffraction Ltd. (Yarnton, UK), was used for unit cell determinations and data reduction. Empirical absorption correction was performed using spherical harmonics, implemented in the SCALE3 ABSPACK scaling algorithm. Crystal structures were solved and refined against all F2 values by using the SHELXTL suite of programs. Hydrogen atoms were placed in calculated positions that were refined using idealised geometries (riding model) and assigned fixed isotropic displacement parameters. All heavy atoms (Gd, W, Mo, Au) were refined anisotropically in all the structures (except the disordered fragments). In addition, non-hydrogen atoms of the frameworks were refined anisotropically in **1** and **1–Mo_6_O_19_** whereas in the other cases these could only be refined isotropically. Distance restraints have been used to model all the triflate anions, some POMs presenting disorder, and some AuCl_4_^−^ anions. In **1**, 3 triflate anions have been refined each with a single thermal parameter, and 1 triflate with partial occupancy fixed to 0.5. In **1–Mo_6_O_19_**, 1 POM unit is disordered over two different sites (with occupancies 59.6(5):40.4(5)) and 1 triflate has been modeled with partial occupancy (0.667(13)). In **1–W_6_O_19_**, 1 POM unit is disordered over two different sites (with occupancies 70.8(4):29.2(4)), which have been modeled with a single thermal parameter; 2 triflate anions has been modeled with partial occupancy fixed to 0.5. In **1–AuCl_4_** 1 AuCl_4_^−^ unit is disordered over two different sites (with occupancies 90:10), and the Cl atoms of the minor components have not been modeled; two AuCl_4_^−^ units have been modeled with partial occupancies fixed to 0.5 and 0.25; six AuCl_4_^−^ fragments have been modeled without the Cl atoms, with partial occupancies of 0.25 (four of them) and 0.125 (two of them); three uncoordinated Cl^−^ anions have been included in the model with partial occupancy fixed to 0.5. In **1–W_6_O_19_–AuCl_4_** 3 POMs have been modeled with partial occupancy (refined to 0.679(5), 0.535(5) and 0.249(6)), with fixed thermal parameters for the oxygen atoms; three AuCl_4_^−^ units have been modeled with partial occupancy (refined to 0.361(5), 0.190(9) and 0.207(5)); two uncoordinated Cl^−^ anions have been included in the model with partial occupancy fixed to 0.5.

All efforts to locate further anions and solvent molecules were unfruitful. The high final R-values are due to large regions, occupied by solvents and anions, which could only be modeled approximately. However, this did not prevent reliable characterization of the cationic framework. A summary of the data collection and structure refinements is provided in Table 1 and Table 2. CCDC-1457100 (**1**), CCDC-1457099 (**1–W_6_O_19_**) CCDC-1457102 (**1–Mo_6_O_19_**), CCDC-1457103 (**1–AuCl_4_**), CCDC-1457101 (**1–W_6_O_19_–AuCl_4_**), contain the supplementary crystallographic data for this paper. These data can be obtained free of charge from The Cambridge Crystallographic Data Center via www.ccdc.cam.ac.uk/data_request/cif.

### 2.3. Powder Diffraction

Polycrystalline samples of **1** and **1–W_6_O_19_** were lightly ground in an agate mortar and pestle and filled into 0.5 mm borosilicate capillaries prior to being mounted and aligned on an Empyrean PANalytical powder diffractometer, using Cu Kα radiation (λ = 1.54056 Å). Two repeated measurements were collected at room temperature (2θ = 5°–30°) and merged in a single diffractogram.

### 2.4. IR

Infrared spectra were recorded in a FT-IR Nicolet 5700 spectrometer (Thermo Scientific, Waltham, MA, USA, EEUU) in the 4000–400 cm^−1^ range using powdered samples diluted in KBr pellets.

### 2.5. EDAX

Metallic composition of bulk samples was estimated by electron probe microanalysis (EPMA) performed in a Philips SEM XL30 (Philips, Amsterdam, Netherlands) equipped with an EDAX microprobe (see Table 3).

## 3. Results and Discussion

### 3.1. Crystal Structure Characterization

The slow layering of a MeOH solution containing 4,4′-bipyridyl-*N,N′*-dioxide (bipyNO) on top of a CH_2_Cl_2_ layer covering the salt [Gd(TfO)_3_]·H_2_O yields crystals of [Gd(bipyNO)_4_]_3_(TfO)_3_·Χ solvent (TfO = triflate). This gadolinium MOF, **1**, is isostructural to other lanthanoid analogues recently reported by us presenting single-ion magnet behavior (SIMMOFs) [24] and crystallizes in the *P* $\bar{1}$ space group, with only minor differences in the unit cell dimensions with the other SIMMOFs due to the different size of the lanthanoid centers. Single crystal analysis reveals the presence of three crystallographically independent Gd ions, which are eight-connected by oxygen atoms belonging to the dioxide linker. Each bipyNO linker forms bridges between two different lanthanoids, with Gd-Gd distances of about 13 Å in the crystal structures, forming a complex 3D network with very large cavities that represent over 70% of the total volume. These cavities are filled with the triflate counterions and solvent molecules, but the large disorder present prevents a complete modeling of all the species located in the voids (see the Experimental Section).

This extremely complex network structure can be classified as a framework of (3^5^4^14^5^9^) (3^5^4^13^5^10^)_2_ topology, comprising a four-connected sub-net of SrAl_2_ topology that is further intersected by two sets of 4^4^ nets that intersect at two distinct eight-connected nodes (with stoichiometry *XY_2_*).

### 3.2. Anion Exchange

**Figure 1:**
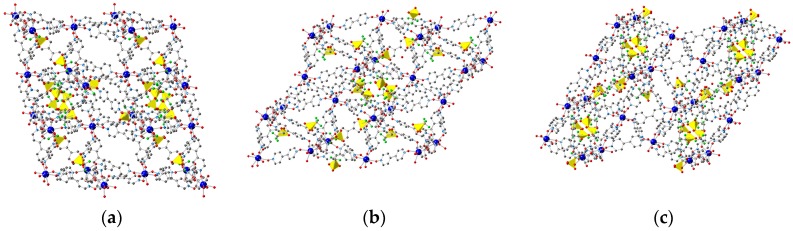
Crystal structure of **1** along the (100), (010) for and (001) directions, shown in (**a**), (**b**) and (**c**) respectively. Color code: Gd, **deep blue**; C, **grey**; N, **blue**; O, **red**; S, **yellow**; F, **green**. Hydrogen atoms omitted for clarity.

The incorporation of new species into the pores of MOFs that permit the integration of additional properties in the material is one of the main applications of these crystalline materials [13]. The presence of triflate anions in the cavities of **1** (Figure 1) makes this MOF potentially attractive for ion-exchange studies. Therefore, we have explored the incorporation of different species in order to understand the ability of the network to incorporate anions of different nature in the porous structure: POMs and other molecular species such as the coordination complex gold tetrachloroaurate. The use of different POMs permits to assess the effects of size and charge in the incorporation process while maintaining the chemical nature of the species, whereas the use of the discrete [AuCl_4_]^−^ metal complex, a molecular precursor of gold nanoparticles, may be of interest for growing these nanoparticles inside the pores of the MOF [82].

**Figure 2:**
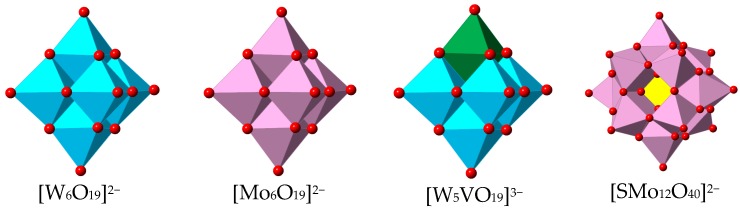
Structural units of the different polyoxometalates (POMs) used for anion exchange studies.

**Figure 3:**
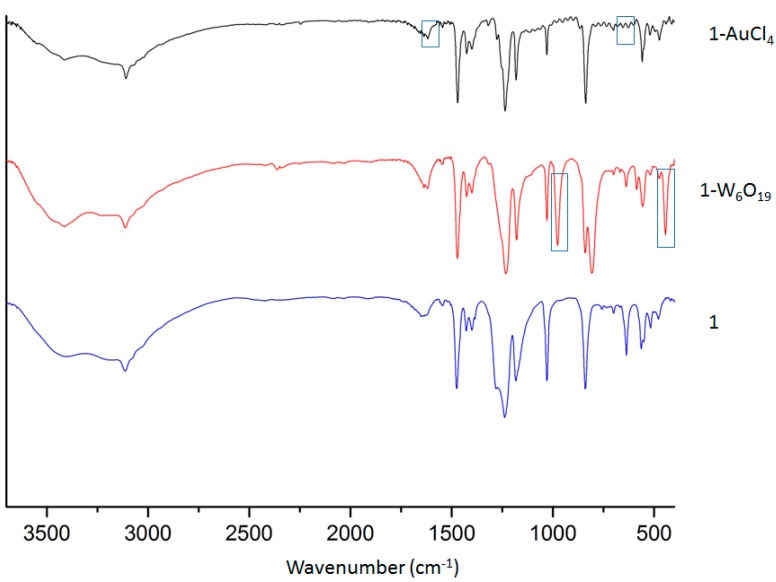
Infrared spectra of pristine **1** and after successful exchange with POMs and [AuCl_4_]^−^. The different boxes highlight the main changes due to the incorporation of the new species.

We have explored the effects of charge and size in the anion exchange process by studying the incorporation in **1** of different types of POMs (Figure 2): [W_6_O_19_]^2−^, [Mo_6_O_19_]^2−^ and [W_5_VO_19_]^3−^ polyanions, all with the Lindqvist structure, and the larger [SMo_12_O_40_]^2−^ polyanion. Crystals of **1** were immersed at room temperature for 1 day in acetonitrile solutions containing the TBA^+^ salts or the (NHex_4_)^+^ of the different POMs. Despite the large size of the POMs, part of the [TfO]^−^ anions were effectively exchanged in the case of the [W_6_O_19_]^2−^ and [Mo_6_O_19_]^2−^ polyanions, which have a diameter of *ca.* 8 Å. The encapsulation of these POMs was clearly observed by infrared spectroscopy (Figure 3) and EDAX, accompanied by a color change of the crystals from colorless to yellow in the case of [Mo_6_O_19_]^2−^ (the crystals remained colorless after exchange with [W_6_O_19_]^2−^). Figure 3 shows the IR spectra of pristine **1** and the exchanged solids. It can be clearly observed the coexistence of the characteristic bands of **1** with those of the POMs. In order to discard the possible presence of POMs and the other species only on the surface of the system, which would also lead to similar IR spectrum, we determined the Gd:S ratio by EDAX (Table 3). Thus, whereas in pristine **1** the Gd:S ratio is 1:3 (as it corresponds to the presence of three triflate anions per Gd), the exchange system **1–W_6_O_19_** shows a Gd:S ratio of 1:1, thus confirming the partial exchange of the triflate anions by POMs. Furthermore, the experimental Gd:W ratio is 1:6, which indicates a composition [Gd(bipyNO)_4_]_3_[W_6_O_19_]_3_(TfO)_3_. Single crystal diffraction analysis (*vide infra*) provides unequivocal evidence of the exchange process.

The inclusion of the POMs in the MOF causes an increase in the structural stability of the open-framework structure when removed from the mother liquor. Thus, whereas the structure of **1** collapses upon solvent removal, the X-ray powder diffraction pattern of **1–W_6_O_19_** shows well-resolved diffraction peaks that perfectly matches with the calculated pattern from the solved structure (Figure S1, Supplementary Material). In contrast, the use of the larger POM [SMo_12_O_40_]^2^^−^ (10.45 Å in size) or one with a larger charge, [W_5_VO_19_]^3^^−^, results in the absence of the characteristic IR signals of the polyanions after all attempts to incorporate them in the cavities of the MOFs (Figure S2, Supplementary Material), thus indicating that both size and charge are important parameters for a successful anion exchange in compound **1**.

In addition to POMs, we have investigated the insertion of [AuCl_4_]^−^. IR analysis reveals the presence of the characteristic bands of these species in the exchanged material (Figure 3), which is further confirmed by EDAX analysis. Interestingly, no S signal is found after the exchange process, revealing a complete anion exchange. Structural characterization of **1–AuCl_4_** has served to obtain crystallographic evidence of the exchange process (*vide infra*). However, all attempts to prepare gold nanoparticles from this encapsulated precursor have resulted unsuccessful so far, likely due to the high dispersity of the anions in the large pores of the MOF.

The different behavior in the anion exchange capabilities of POMs and [AuCl_4_]^−^, being complete only for [AuCl_4_]^−^, is likely due to the smaller size of the [AuCl_4_]^−^ anion compared to the POMs. This means that compound **1** has accessible voids of different sizes that could permit the co-existence of both molecular species in a single material, with the POMs filling the large voids and the [AuCl_4_]^−^ filling the small ones. This was achieved in a two-step procedure: first, all the [TfO]^−^ anions were successfully exchanged with [AuCl_4_]^−^, resulting in **1–AuCl_4_**. This exchanged solid was subsequently exposed to [W_6_O_19_]^2^^−^ anions. As expected, this second exchange process was not complete, similarly to the direct exchange of the triflate anions in **1** with POMs. EDAX analysis shows the co-existence of Gd, Au, Cl and W, and a complete absence of S. Single crystal diffraction served to unequivocally prove the presence of [AuCl_4_]^−^ and [W_6_O_19_]^2^^−^ in the cavities (*vide infra*).

### 3.3. Location of Interchanged Anions

**Figure 4:**
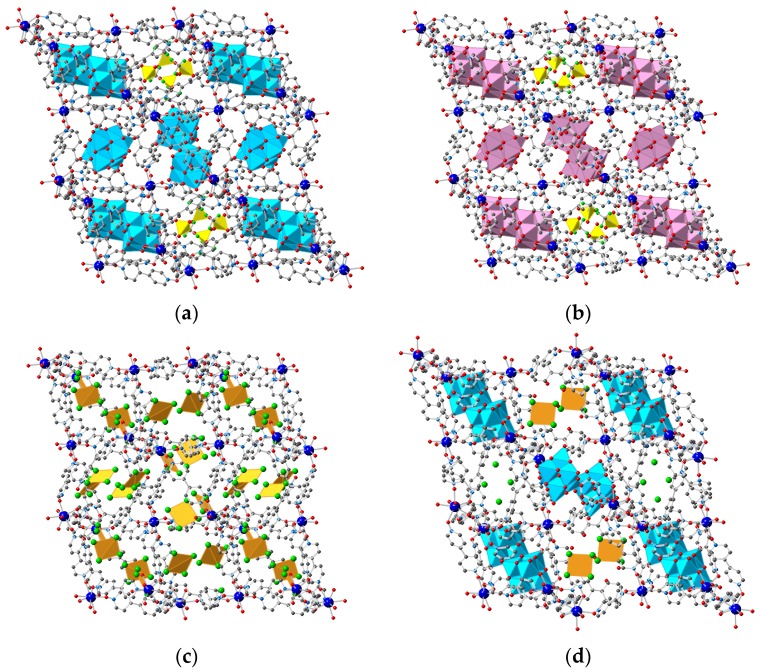
Crystal structures of the anion exchanged solids: (**a**) **1–W_6_O_19_**; (**b**) **1–Mo_6_O_19_**; (**c**) **1–AuCl_4_** and (**d**) the mixed system **1–W_6_O_19_–AuCl_4_**. Color code of the framework as in Figure 1. [W_6_O_19_]^2−^ [Mo_6_O_19_]^2−^, [AuCl_4_]^−^ and Cl^−^ anions shown in **blue**, **purple**, **orange** and **green**, respectively.

The successful anion exchange process has been unequivocally demonstrated by X-ray single crystal diffraction of the exchanged products **1–W_6_O_19_**, **1–Mo_6_O_19_**, **1–AuCl_4_** and the mixed system **1–W_6_O_19_–AuCl_4_**. In the case of **1–W_6_O_19_** and **1–Mo_6_O_19_** the anion exchange process was not complete and some [TfO]^−^ anions were also observed, as confirmed by EDAX (see Figure 4 and Figures S3–S5, Supplementary Material).

**Figure 5:**
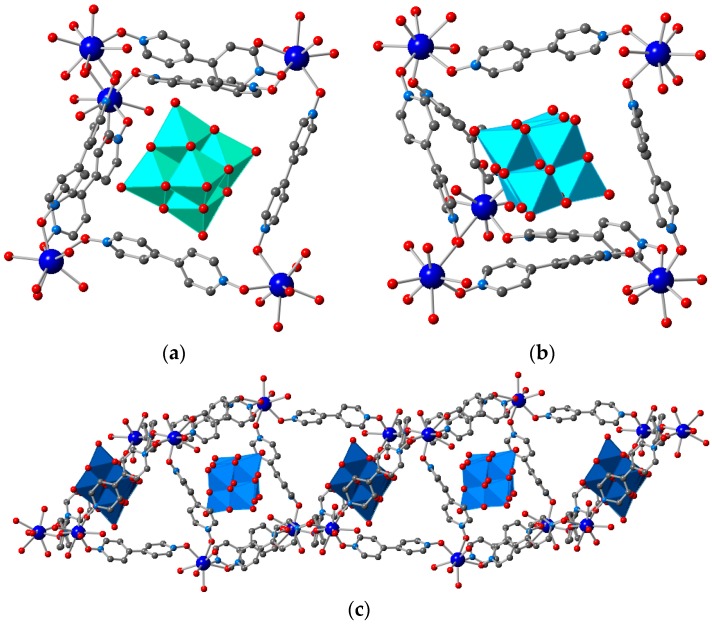
Different environments (**a**, **b**, and **c**) of the four crystallographically independent [W_6_O_19_]^2−^ anions after anion exchange (similar results are obtained for [Mo_6_O_19_]^2−^).

In both **1–Mo_6_O_19_** and **1–W_6_O_19_**, three POM units are incorporated per asymmetric unit of the MOF, thus resulting in a compound of stoichiometry [Gd(bipyNO)_4_]_3_(POM)_3_(TfO)_3_. The two compounds present the POM^2−^ anions located in the same positions of the structure, which correspond to large cavities, far from the Ln centers (the shortest Ln···O(POM) distance are 5.315 and 5.350 Å for **1–Mo_6_O_19_** and **1–W_6_O_19_**, respectively) and forming weak interactions with the framework through weak C–H···O hydrogen bonds. Two of the POMs are located at inversion centers, and thus there is a total of two complete and two halves crystallographycally independent POMs. Figure 5 shows the environment of the four different POM anions. Interestingly, one of the POMs presents disorder in the two different crystal structures, thus confirming the weakness of their interaction with the framework. Importantly, the structural features of the network remain unchanged upon anion exchange, despite the bulkiness of the POM^2−^ anions.

**Figure 6:**
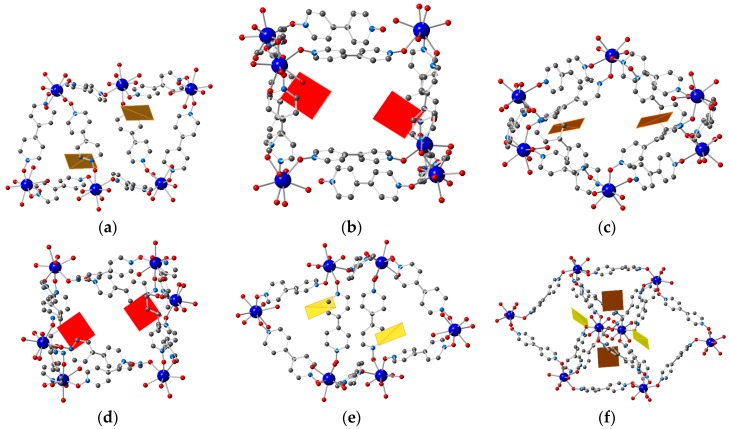
Different environments of the crystallographically independent [AuCl_4_]^−^ anions after anion exchange (**a**–**f**).

**Figure 7:**
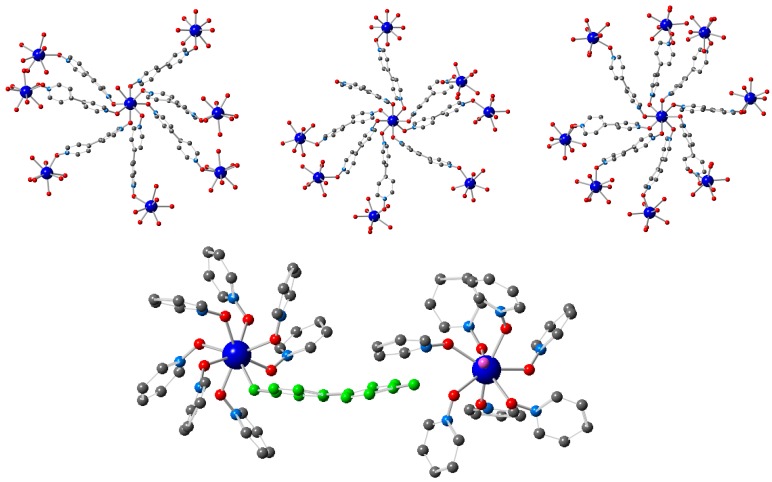
(**Top**) View of the three crystallographically independent Gd centers in **1–AuCl_4_**: Gd1, Gd2 and Gd3. Gd1 has a coordinated water molecule (in addition to 7 bridging bipyNO ligands), Gd2 has a terminal bipyNO ligand (in addition to 7 bridging bipyNO ligands), and Gd3 is coordinated to 8 bipyNO ligands that serve as bridges between Gd centers. (**Bottom**) Close view of the coordination environment of Gd1 and Gd2, highlighting the presence of a terminal bipyNO ligand (in **green**) and a coordinated water molecule (in **pink**).

In **1–AuCl_4_** 7.5 AuCl_4_^−^ and 1.5 Cl^−^ anions are incorporated per asymmetric unit of the MOF, *i.e.*, all the nine triflate anions have been exchanged, as established by EDAX. Therefore, the resultant stoichiometry of the exchanged material is [Gd(bipyNO)_4_]_3_(AuCl_4_)_7.5_(Cl)_1.5_. Figure 6 shows the different environment of the crystallographically different AuCl_4_^−^ anions. These anions form weak interactions with the framework through weak C–H···Cl hydrogen bonds [83]. Interestingly, contrary to what is observed in the exchange process with POMs, there are some modifications in the structural features of the framework upon anion exchange. Specifically, one of the bipyNO ligands is no longer coordinated to two Gd centers, but is only bound to a single Gd. The coordination sphere of the Gd center, which is now coordinated to seven bipyNO ligands, is completed with a water molecule (Figure 7). In addition, there is a minor change in the unit cell dimensions, with a larger value for α (see Table 1 and Table 2 for crystallographic information).

Finally, the possible inclusion of two different anions was also examined. The totally different structures of [W_6_O_19_]^2^^−^ and [AuCl_4_]^−^ allows an easy discrimination between them through X-ray analysis. Examination of the singly exchanged structures **1–W_6_O_19_**, and **1–AuCl_4_** reveals that there are some areas of the MOFs which can be loaded with [AuCl_4_]^−^ anions but are not accessible to [W_6_O_19_]^2^^−^ (compare Figure 4a,c), thus suggesting a possible selectivity in a mixed anion exchange process, which has been unequivocally proved by single crystal diffraction (Figure 4d). The [W_6_O_19_]^2^^−^ anions are located in the same regions of the MOF as observed in the singly exchanged material **1–W_6_O_19_**, whereas the [AuCl_4_]^−^ anions are located where the triflate anions were observed. The reverse procedure, *i.e.*, the initial exchange with POM and subsequent exchange with [AuCl_4_]^−^, was unsuccessful for the coexistence of the two species in the cavities, as all the POMs that are initially exchanged suffer a complete anion exchange process resulting in **1–AuCl_4_**.

## 4. Conclusions

In this work, we have conducted a detailed analysis of the anion exchange capabilities of a Gd MOF through investigation of the effects of size and charge in the process. Anion exchange process is a post-synthetic modification that allows the incorporation of interesting species like polyoxometalates or other metal-organic species. POMs [W_6_O_19_]^2−^ and [Mo_6_O_19_]^2−^, with the Lindqvist structure and a size of *ca.* 8 Å, can be incorporated into the pores of the MOFs in a single-crystal-to-single-crystal manner, although the exchange is not complete and triflate anions of the original material remain in the pores. The increase of the charge of the POM maintaining the size (through the use of the Lindqvist anion [W_5_VO_19_]^3−^) or the increase of the size of the POM maintaining the charge (through the use of [SMo_12_O_40_]^2−^, of 10.5 Å in size) results in an unsuccessful anion exchange. In contrast, use of smaller anionic species such as [AuCl_4_]^−^ allows a complete exchange process with no triflate anions remaining in the interior of the MOF.

Furthermore, we have proved the possibility of performing a two-step exchange in order to incorporate different anions that can co-exist in the same material. This feature can have potential applications for the inclusion of other non-innocent anions to obtain synergistic effects. The success of the anion exchange process has been determined easily by combination of IR spectroscopy and EDAX analysis, but more detailed and accurate information has been provided by single-crystal analysis.

This approximation opens the path for the incorporation of other metalate complexes that could also react after the inclusion in the pores of the MOFs in order to form polynuclear structures in a ship-in-a-bottle procedure.

## Figures and Tables

**Table 1:** Crystallographic data for compounds **1**, **1–W_6_O_19_** and **1–Mo_6_O_19_**.

Compound	1	1–W_6_O_19_	1–Mo_6_O_19_
Empirical formula	C_129_H_96_F_27_Gd_3_N_24_O_51_S_9_	C_123_H_96_F_9_Gd_3_N_24_O_90_S_3_W_18_	C_123_H_96_F_9_Gd_3_Mo_18_N_24_O_90_S_3_
Formula weight	4,071.59	7,398.47	5,816.09
Crystal color	Colorless	Colorless	Yellow
Crystal size (mm^3^)	0.13 × 0.11 × 0.06	0.10 × 0.08 × 0.06	0.17 × 0.11 × 0.05
Temperature (K)	120(2)	120(2)	120(2)
Crystal system, Z	Triclinic, 2	Triclinic, 2	Triclinic, 2
Space group	*P* $\bar{1}$	*P* $\bar{1}$	*P* $\bar{1}$
*a* (Å)	24.0370(8)	24.0555(13)	24.0547(9)
*b* (Å)	24.0747(8)	24.0908(6)	24.1561(9)
*c* (Å)	24.4414(7)	24.6766(8)	24.6456(6)
*α* (°)	85.291(2)	86.126(2)	86.253(2)
*β* (°)	62.188(3)	61.429(4)	61.542(3)
*γ* (°)	61.627(3)	61.276(4)	61.515(4)
*V* (Å^3^)	10,828.6(6)	10,766.3(7)	10,820.5(6)
ρ_calc_ (mg/m^3^)	1.249	2.282	1.785
μ(Mo_Kα_) (mm^−1^)	1.085	10.600	2.030
θ range (°)	3.24–25.05	2.91–25.04	2.85–25.06
Reflns collected	80,917	83,116	186,594
Independent reflns (*R*_int_)	38,196(0.0717)	37,930(0.1141)	38,229(0.1116)
Reflns used in refinement, *n*	38,196	37,930	38,229
L. S. parameters, *p*/restraints, *r*	1,659/74	864/198	1,841/128
*R*1(*F*),^a^ *I* > 2σ(*I*)	0.1347	0.1509	0.1687
*wR*2(*F*^2^),^b^ all data	0.4321	0.4559	0.4969
*S*(*F*^2^),^c^ all data	1.503	1.244	1.866

^a^ *R1*(*F*) = Σ(|*Fo*| − |*Fc*|)/Σ|*Fo*|; ^b^ *wR*2(*F*^2^) = [Σ*w*(*Fo*^2^ − *Fc*^2^)^2^/Σ*wFo*^4^]^½^; ^c^ *S*(*F*^2^) = [Σ*w*(*Fo*^2^ − *Fc*^2^)^2^/(*n* + *r −p)*]^½^.

**Table 2:** Crystallographic data for compounds **1–AuCl_4_** and **1–W_6_O_19_–AuCl_4_**.

Compound	1–AuCl_4_	1–W_6_O_19_–AuCl_4_
Empirical formula	C_120_H_96_Au_7.50_Cl_31.50_Gd_3_N_24_O_25_	C_120_H_96_Au_1.50_Cl_9_Gd_3_N_24_O_66.25_W_13.5_
Formula weight	5,339.88	6,510.43
Crystal color	Yellow	Yellow
Crystal size (mm^3^)	0.07 × 0.07 × 0.05	0.08 × 0.06 × 0.06
Temperature (K)	120(2)	120(2)
Crystal system, Z	Triclinic, 2	Triclinic, 2
Space group	*P* $\bar{1}$	*P* $\bar{1}$
*a* (Å)	23.9544(4)	23.8865(16)
*b* (Å)	24.3447(4)	24.1640(15)
*c* (Å)	24.8313(4)	24.5613(16)
*α* (°)	71.2790(10)	86.866(5)
*β* (°)	62.647(2)	61.457(7)
*γ* (°)	60.725(2)	61.595(6)
*V* (Å^3^)	11,132.0(3)	10,677.4(12)
ρ_calc_ (mg/m^3^)	1.593	2.025
μ(Mo_Kα_) (mm^−1^)	6.229	9.364
θ range (°)	3.23–25.08	3.27–25.05
Reflns collected	241,553	131,931
Independent reflns (*R_int_*)	39,355(0.1234)	37,696(0.2606)
Reflns used in refinement, *n*	39,355	37,696
L. S. parameters, *p*/restraints, *r*	910/24	681/98
*R*1(*F*),^a^ *I*>2*σ*(*I*)	0.1489	0.2284
*wR*2(*F*^2^),^b^ all data	0.4541	0.5898
*S*(*F*^2^),^c^ all data	1.768	1.482

^a^ *R1*(*F*) = Σ(|*Fo*| − |*Fc*|)/Σ|*Fo*|; ^b^ *wR*2(*F*^2^) = [Σ*w*(*Fo*^2^ − *Fc*^2^)^2^/Σ*wFo*^4^]^½^; ^c^ *S*(*F*^2^) = [Σ*w*(*Fo*^2^ − *Fc*^2^)^2^/(*n* + *r* − *p)*]^½^.

**Table 3:** Ratio determination for Gd, S, W, Au by EDAX (T: Theoretical, E: Experimental).

	1 (T)	1 (E)	1–W_6_O_19_ (T)	1–W_6_O_19_ (E)	1–AuCl_4_ (T)	1–AuCl_4_ (E)	1–W_6_O_19_-AuCl_4_ (T)	1–W_6_O_19_–AuCl_4_ (E)
Gd	1	1	1	1.2	1	1	1	2.4
S	3	3.4	1	1.0	–	–	–	–
W	–	–	6	6.02	–	–	6	10
Au	–	–	–	–	3	3.12	1	1

## Supplementary Materials

Supplementary materials can be found at www.mdpi.com/2073-4360/8/5/171/s1.

## Abbreviations

The following abbreviations are used in this manuscript:

MOF	Metal organic framework
POM	Polyoxomatalate
TfO	Triflate
MeOH	Methanol
