# Supplementary material for: Single-Crystal-to-Single-Crystal Anion Exchange in a Gadolinium MOF: Incorporation of POMs and [AuCl4]−
Source: Polymers (Basel). 2016 Apr 26;8(5):171. doi: 10.3390/polym8050171 (PMC6431858; doi:10.3390/polym8050171)
Supplement: Supplementary file 1 [file polymers-08-00171-s001.pdf]

Supplementary Materials: Single-Crystal-to-Single-Crystal Anion Exchange in a Gadolinium MOF: Incorporation of POMs and $[AuCl_4]^-$

Javier López-Cabrelles, Guillermo Mínguez Espallargas * and Eugenio Coronado

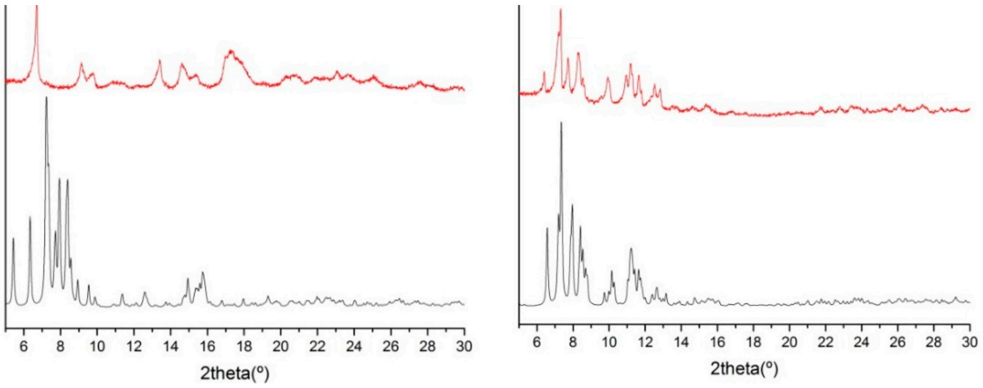

Figure S1. X-ray powder patterns of **1** (**left**) and **1–W_6O_{19}**, (**right**). The experimental patterns are shown in red and the calculated pattern from single crystal data are shown in black. It can be clearly seen that whereas the structure of 1 collapses upon solvent removal, **1–W_6O_{19}**, remains stable.

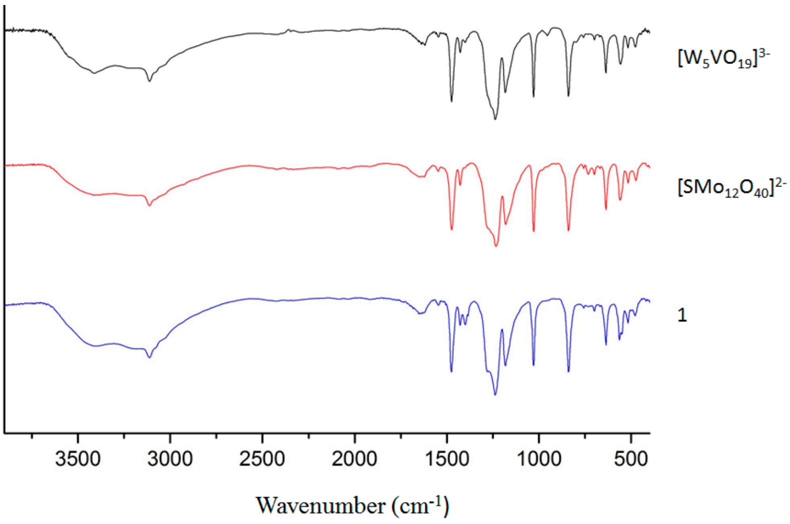

Figure S2. Infrared spectra of pristine **1** and after the unsuccessful anion exchange with $[SMo_{12}O_{40}]^{2-}$ and $[W_5VO_{19}]^{3-}$ POMs.

100

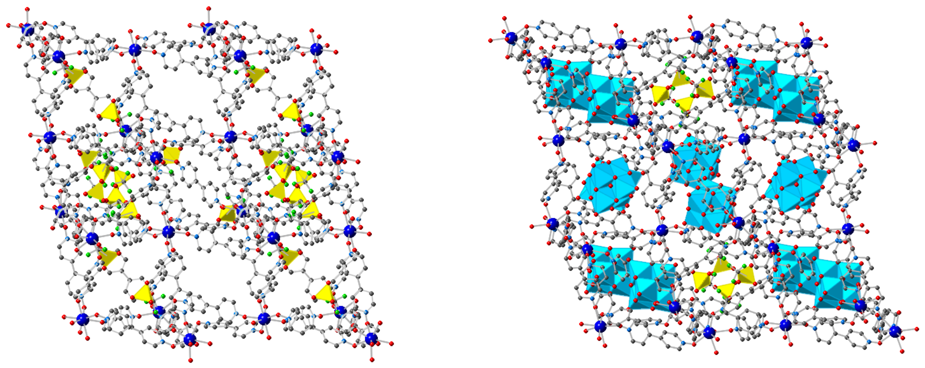

Figure S3. *Cont.*

010

001

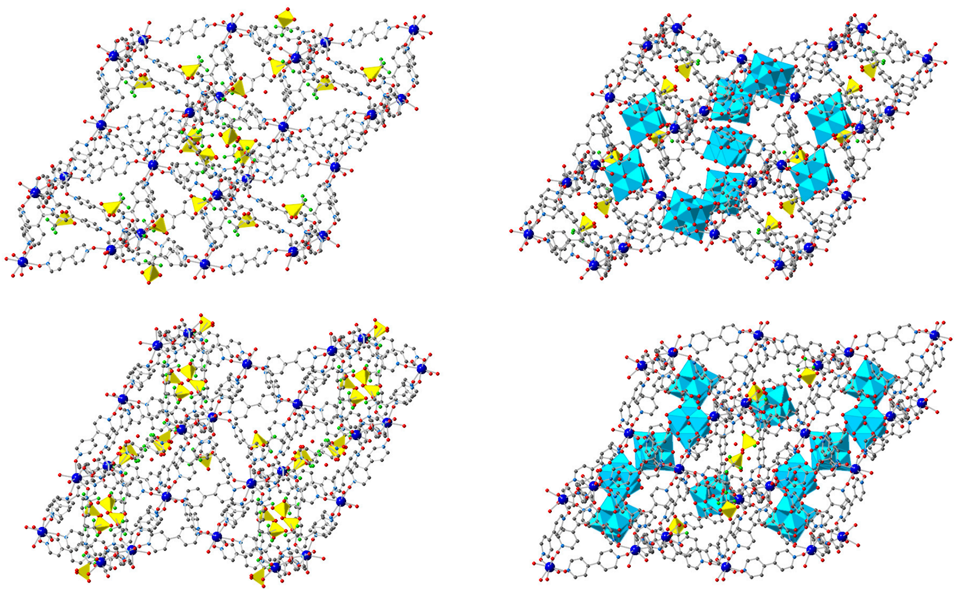

Figure S3. Views along the (100), (010) and (001) directions (**left**, **middle** and **right**, respectively) of the crystal structures of **1** and **1–W_6O_{19}**.

100

010

001

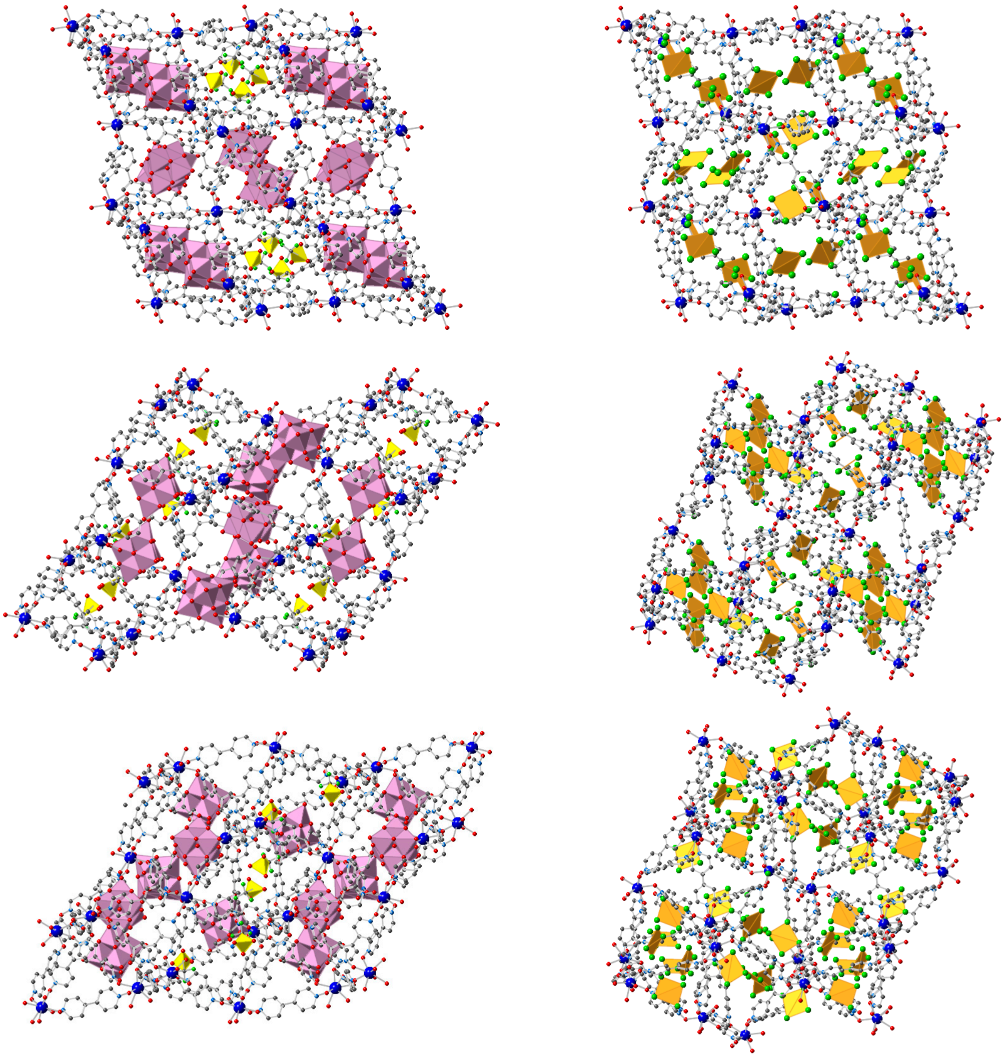

Figure S4. Views along the (100), (010) and (001) directions (**left**, **middle** and **right**, respectively) of the crystal structures of **1-Mo_6O_{19}** and **1-$AuCl_4$**.

100

010

001

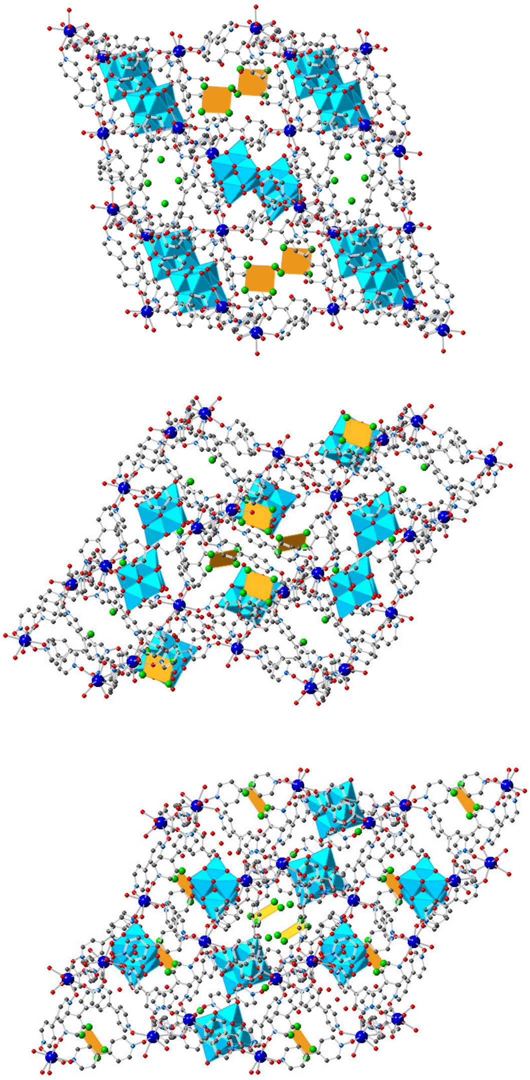

Figure S5. Views along the (100), (010) and (001) directions (**left**, **middle** and **right**, respectively) of the crystal structure of **1–W_6O_{19}–$AuCl_4$**.

© 2016 by the authors; licensee MDPI, Basel, Switzerland. This article is an open access article distributed under the terms and conditions of the Creative Commons Attribution (CC-BY) license (http://creativecommons.org/licenses/by/4.0/).
